# An Elucidation of Neutrophil Functions against *Mycobacterium tuberculosis* Infection

**DOI:** 10.1155/2013/959650

**Published:** 2013-11-07

**Authors:** Devin Morris, Thien Nguyen, John Kim, Christine Kassissa, Melissa Khurasany, Jennifer Luong, Sarah Kasko, Shalin Pandya, Michael Chu, Po-Ting Chi, Judy Ly, Minette Lagman, Vishwanath Venketaraman

**Affiliations:** ^1^College of Osteopathic Medicine of the Pacific, Western University of Health Sciences, Pomona, CA 91766, USA; ^2^College of Dental Medicine, Western University of Health Sciences, Pomona, CA 91766, USA; ^3^Graduate College of Biomedical Sciences, Western University of Health Sciences, Pomona, CA 91766, USA

## Abstract

We characterized the functions of neutrophils in response to *Mycobacterium tuberculosis* (*M. tb*) infection, with particular reference to glutathione (GSH). We examined the effects of GSH in improving the ability of neutrophils to control intracellular *M. tb* infection. Our findings indicate that increasing the intracellular levels of GSH with a liposomal formulation of GSH (L-GSH) resulted in reduction in the levels of free radicals and increased acidification of *M. tb* containing phagosomes leading to the inhibition in the growth of *M. tb*. This inhibitory mechanism is dependent on the presence of TNF-**α** and IL-6. Our studies demonstrate a novel regulatory mechanism adapted by the neutrophils to control *M. tb* infection.

## 1. Introduction

*M. tb*, a causative agent for tuberculosis (TB), is known to be a highly successful intracellular pathogen and is estimated to be harbored in one third of the world's population [1]. Left untreated TB is known to have a 70% mortality rate [2]. Increasing drug resistance strains of *M. tb* and poor precautionary measurements for containing the spread, especially in “high burden” countries, have kept the disease from global eradication [1]. *M. tb* infection is mostly confined to lungs and can be transmitted by inhalation of droplets from individuals with active TB. *M. tb *has the ability to evade the host killing mechanisms and can successfully persist inside the phagocytic cells. Specifically, *M. tb *is capable of surviving within the host cells by preventing the fusion of the phagosomes containing bacteria with lysosomes, thereby avoiding exposure to the toxic lysosomal hydrolases [3].

The terminally differentiated polymorphonuclear leukocytes (PMN) or neutrophils are essential component of the human innate immune system and act as a first-line defense against invading microorganisms. Neutrophils are major inhabitants of the white blood cell population (WBC) since they constitute ~70% of the total WBC population [4]. Neutrophils form the bulk of early recruited leukocyte population that mediate and elicit immune responses against *M. tb *[4]. Neutrophils, after encountering the mycobacteria, exhibit innate immune responses such as phagocytosis which in turn triggers signaling events resulting in secretion of cytokines such as TNF-*α* and IL-1 that mediate early inflammatory responses [4–6].

The tripeptide, glutathione (GSH), protects all cells against oxidizing agents, free radicals, and reactive oxygen intermediates (ROI), either directly or through enzymatic action of GSH peroxidases and GSH-transferases [7, 8]. GSH is also important for cellular homeostasis as well as many different cellular functions such as protein synthesis, enzyme catalysis, transmembrane transport, receptor action, intermediary metabolism, and cell maturation [9–11]. GSH is produced by nearly all cell types and exists in two forms: reduced or free GSH (*r*GSH) and oxidized (GSSG). *r*GSH is responsible for the antioxidant functions of GSH, while GSSG is the byproduct of the free radical scavenging activity of GSH and lacks antioxidant function [9–11].

We have previously reported that GSH has antimycobacterial effects and furthermore enhancing the levels of GSH in human macrophages resulted in inhibition in the growth of intracellular *M. tb* [12]. Additionally, increasing the levels of GSH in immune cells, such as natural killer (NK) cells and T cells, resulted in improved control of *M. tb* infection inside human monocytes indicating that GSH has both direct antimycobacterial effects as well as immune enhancing effects [13, 14].

Though many studies have highlighted the roles of monocytes and macrophages in innate immune responses against *M. tb* infection, very few studies have demonstrated the importance of neutrophils during *M. tb* infection [5, 6]. The role of GSH in regards to the neutrophil innate response and pathophysiology has been poorly defined. In this study, an attempt was made to investigate the role of neutrophils in innate defense against *M. tb* infection.

We chose to examine the functions of neutrophils against *M. tb* infection for three chief reasons: first, they mirror macrophage mechanisms mainly by phagocytosing bacteria, second, they are one of the first responders to bacterial infections, and third, they are closely tied to free radical and inflammatory responses. We also characterized the underlying mechanisms by which GSH-enhanced neutrophils are able to successfully inhibit the growth of *M. tb*. We hypothesized that augmenting the levels of GSH in neutrophils will increase the fusion of phagosomes containing *M. tb* with lysosomes leading to inhibition in the growth of *M. tb*. The ultimate purpose of this research is to determine the ability of GSH-enhanced neutrophils to effectively control *M. tb* infection. By better characterizing the role of neutrophils which form a major population of WBC, we can further advance the crusade against *M. tb* control and eradication.

We tested our hypothesis by examining the effects of two different GSH-enhancing agents: a GSH precursor, N-acetyl cysteine (NAC), and L-GSH, a liposomal formulation of GSH, in mediating the control of *M. tb* infection inside neutrophils. We correlated the inhibition in the growth of *M. tb* inside neutrophils with the levels of GSH and free radicals, production of inflammatory cytokines such as IL-6 and TNF-*α*, and the extent of phagosome lysosome fusion. Our results indicate that treatment of neutrophils with L-GSH resulted in inhibition in the growth of *M. tb* and this inhibition was accompanied by increased fusion between phagosomes containing *M. tb *with lysosomes. Our findings unveiled one of the important mechanisms by which GSH improves the ability of neutrophils to control *M. tb *infection.

## 2. Materials and Methods

### 2.1. Statement of Ethics

All studies were approved by both the Institutional Review Board and the Institutional bio-safety committee of the Western University of Health Sciences. All study participants were above the legal age of consent at the time of participation, and written informed consent was obtained from all volunteers prior to participation in the study.

### 2.2. Subjects

Healthy subjects without a history of TB were recruited from the students, faculty, and staff of Western University of Health Sciences. Forty milliliters of blood was drawn once from each healthy volunteer after obtaining signed informed consent.

### 2.3. Isolation of Neutrophils

Neutrophils were isolated from the whole blood of healthy individuals using density gradient centrifugation with dextran and Ficoll histopaque, a high density pH neutral polysaccharide solution (Sigma). Briefly, 40 milliliters of whole blood was diluted in equal volume of 0.9% NaCl saline solution (1 : 1), mixed gently, layered on top of the Ficoll-hypaque, and centrifuged for duration of 40 minutes at 1400 rpm. The neutrophils rich RBC layer was diluted to a total volume of 25 mL with hanks balanced salt solution, mixed with equal volume of 6% dextran (specific average molecular weight of 250,000 g), and incubated for 2 hours to allow for gravitational sedimentation of RBCs. Following incubation, the semitransparent supernatant was aspirated off and centrifuged for 10 minutes at 1400 rpm. Subsequent to centrifugation, the soft pellet containing PMN's and RBC's was gently dispersed and resuspended in PBS. Cells were washed once in PBS, and the contaminating RBC's were eliminated by treating the cell suspension with RBC lysis buffer. Purified neutrophils were resuspended in Roswell Park Memorial Institute (RPMI) medium containing 5% AB serum. 1 × 10^4^ neutrophils were added to each well of the polylysine coated 96-well tissue culture plates and incubated for 12 hours at 37°C, 5% CO_2_ incubator for adherence. Following overnight adherence, nonadherent cells were removed by washing, and the adherent neutrophils were resuspended in fresh media and used for various assays. Purity of the cultures was determined by microscopic examination of nuclear morphology after staining with 4′, 6-diamidino-2-phenylindole (DAPI).

### 2.4. Preparation of Bacterial Cells for Neutrophil Infection

All infection studies were performed using the virulent laboratory strain of *M. tb*, H37Rv, inside the biosafety level 3 (BSL-3) facility. *M. tb, *H37Rv was processed for infection as follows: static cultures of H37Rv at their peak logarithmic phase of growth (between 0.5 and 0.8 at A600) were used for infection of neutrophils. The bacterial suspension was washed and re-suspended in RPMI (Sigma) containing AB serum (Sigma). Bacterial clumps were disaggregated by vortexing five times with 3 mm sterile glass beads. The bacterial suspension was passed through a 5 *μ*m syringe filter (Millipore) to remove any existing clumps. The total number of organisms in the suspension was ascertained by plating. Processed H37Rv were frozen as stocks at −80°C. At the time of infection, frozen stocks of processed H37Rv were thawed and used for infection of neutrophils.

### 2.5. Infection of Neutrophils with H37Rv

To examine the role of neutrophils and GSH in innate defense against *M. tb* infection, we determined the intracellular survival of *M. tb* inside neutrophils that were cultured in the presence and absence of GSH-enhancing agents. Neutrophils were infected with processed H37Rv at a low dose multiplicity of infection of 1 : 10 (1 bacterium for every 10 neutrophils) and incubated for 2 hours to allow for phagocytosis. Un-phagocytosed bacteria were removed by washing the infected neutrophil cultures three times with warm sterile PBS (based on the results from our standardization studies, this low dose multiplicity of infection usually resulted in approximately 10% of the neutrophils infected with H37Rv, and each infected cell had between 1 and 5 bacteria). Infected neutrophils were cultured in RPMI + 5% AB serum at 37°C + 5% CO_2_ in the presence and absence of NAC (10 mM) or L-GSH (10 and 20 *μ*M, Your Energy Systems, LLC Palo Alto, CA). The liposomal formulation of GSH (L-GSH) used in these studies is a proprietary product of Your Energy Systems (YES), LLC (Palo Alto, CA) called ReadiSorb. Infected neutrophils were terminated at 1 hour and 24 hours after infection to determine the intracellular survival of H37Rv.

### 2.6. Termination of Infected Neutrophils and Measurement of Colony Forming Units (CFU)

Termination of H37Rv-infected neutrophils was performed by the addition of 200 *μ*L of sterile distilled water to each culture well. The collected neutrophil lysates were diluted in sterile water and plated on 7H11 medium (Hi Media) enriched with albumin dextrose complex (ADC) to estimate the extent of H37Rv growth in neutrophils by counting the CFUs.

### 2.7. Neutrophil Viability Assay

The effects of GSH-enhancing agents in altering the viability of H37Rv-infected neutrophils were determined by fluorescent DAPI staining. Neutrophils (1 × 10^4^/well) were distributed in sterile glass cover slips positioned in 24-well tissue culture plates and incubated for 12 hours for adherence. Adhered neutrophils were infected H37Rv at a multiplicity of infection of 1 : 10 (1 bacteria to 10 neutrophils). Infected neutrophils were cultured in RPMI + 5% AB serum at 37°C + 5% CO_2_ in the presence and absence of NAC (10 mM) or L-GSH (10 and 20 *μ*M) for 24 hours. Infected neutrophils were fixed with 3.8% paraformaldehyde in PBS for 30 minutes. Fixed neutrophils were washed twice for 5 minutes in PBS. A single drop of mounting media containing DAPI was placed per glass slide. The stained neutrophils were then attached to glass cover slips and inverted onto the glass slide. The cover slips were then sealed to the glass slide by smearing nail polish around the edges of the cover slips. The stained slides were then viewed using an inverted fluorescent microscope. Intact cells with DAPI staining were counted under 20x magnification in 7 randomly selected fields for each category. A neutrophil survival index was generated by dividing the number of surviving neutrophils in each treatment category by the number of surviving neutrophils in the untreated control category.

### 2.8. Assay of GSH Levels in Neutrophils

GSH levels in neutrophils were measured by spectrophotometry using an assay kit from Arbor Assays. Briefly, uninfected and H37Rv-infected neutrophils (1 × 10^4^) were lysed by the addition of ice cold 5% 5-sulfosalicylic acid dehydrate solution (MP Biomedicals). Lysates were centrifuged, and supernatants collected after centrifugation were analyzed for total GSH and oxidized GSH (GSSG) as per manufacturer's instructions. Levels of free or reduced GSH (*r*GSH) were calculated by subtracting measured GSSG concentrations from the measured total GSH concentrations. All GSH measurements were normalized with total protein levels.

### 2.9. Assay of Total Protein Levels in Lysates

Proteins in the isolated cell lysates were measured by Bradford's method using a Coomassie protein assay reagent (Thermo Scientific). This assay helped to determine the amount of protein in each well. By dividing the total protein value from the GSH assay, it helped to confirm that the variation of GSH is due to the treatments added.

### 2.10. Assay of IL-6 and TNF-*α* in Neutrophil Supernatants

Levels of IL-6 and TNF*-*α**in supernatants from uninfected and H37Rv-infected neutrophils were measured by enzyme linked immunosorbent assay (ELISA) (eBioscience). 

### 2.11. Assay of Free Radicals in Neutrophil Lysates

Free radical levels in neutrophil lysates were determined by measuring the levels of malondialdehyde (MDA) using a colorimetric assay kit from Cayman. The MDA levels in the infected groups (*M. tb*-infected, *M. tb*-infected and treated with NAC, and *M. tb*-infected and treated with L-GSH) were normalized to the values in uninfected neutrophils. 

### 2.12. Fluorescent Microscopic Analysis of *M. tb* Phagosome Acidification in Neutrophils

The effects of GSH-enhancing agents in inducing acidification of *M. tb*-containing phagosomes were determined by quantifying the colocalization of Fluorescein Isothiocyanate (FITC) labeled H37Rv in the lysotracker labeled acidified compartments by fluorescent microscope. Lysotracker Red DND99 (Molecular Probes), an acidotropic dye, is a weak base conjugated to a red fluorophore. Lysotracker freely permeates cell membranes and gets trapped upon protonation in an acidified compartment. The excitation and emission maxima of lysotracker are 577 and 592 nm, respectively. Neutrophils (1 × 10^4^/well) were distributed in polylysine coated sterile glass cover slips positioned in 24-well tissue culture plates and incubated for 12 hours for adherence. Adhered neutrophils were treated with lysotracker (1/10^3^ dilution) for 1 hour. H37Rv was labeled with FITC as follows; bacterial cells were incubated with 0.8 mg/mL FITC for 1 hour. The labeled cells were then centrifuged, and the pellet was resuspended in PBS and used for infecting the lysotracker-labeled neutrophils. Lysotracker-labeled neutrophils were incubated with FITC-labeled H37Rv for 1.5 hours to allow phagocytosis. Infected neutrophils were cultured in RPMI + 5% AB serum at 37°C + 5% CO_2_ in the presence and absence of NAC (10 mM) or L-GSH (10 and 20 *μ*M) for 24 hours. Infected neutrophils were fixed with 3.8% paraformaldehyde in PBS for 30 minutes. Fixed neutrophils were washed twice for 5 minutes in PBS. A single drop of mounting media containing DAPI was placed per glass slide. The stained neutrophils were then attached to glass cover slips and inverted onto the glass slide. The cover slips were then sealed to the glass slide by smearing nail polish around the edges of the cover slips. The stained slides were then viewed using an inverted fluorescent microscope. Images were obtained using an integrated digital camera. Images were subsequently analyzed using ImageJ, a free software program available from the National Institutes of Health (http://rsbweb.nih.gov/ij/). Following background fluorescence correction, the average fluorescent intensity was measured.

### 2.13. Statistical Analysis

All statistical analysis was done using Graph Pad Prism6 software on the mean ± standard error for *n* = 5 individuals. For all assays, the infected-untreated category was compared to the uninfected-untreated control category using a two-tailed Student's *t*-test with 95% confidence intervals. All infected-treated categories were compared to the infected-untreated category using a Kruskal-Wallis 1-way ANOVA, with Dunn's multiple comparisons posttest. Results were considered significant for *P* ≤ 0.05.

## 3. Results

### 3.1. Intracellular Survival of H37Rv inside Human Neutrophils

**Figure 1:**
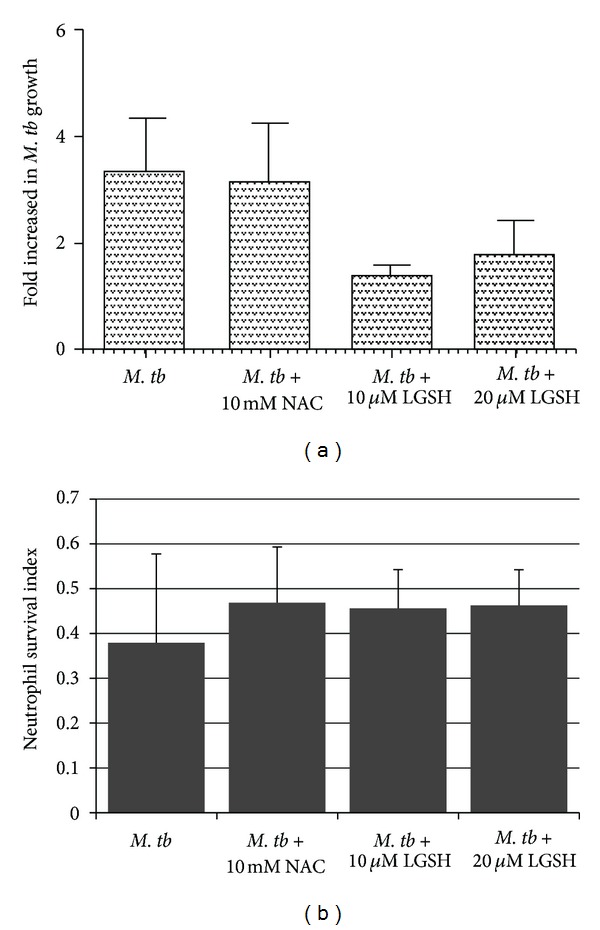
*Intracellular survival of H37Rv in neutrophils treated with NAC and L-GSH (n* = 5*).* Neutrophils were infected with processed H37Rv at a low dose multiplicity of infection of 1 : 10 (1 bacterium for every 10 neutrophils) and incubated for 2 hours to allow for phagocytosis. Unphagocytosed mycobacteria were removed by washing the infected neutrophils three times with warm sterile PBS. Infected neutrophils were cultured in RPMI + 5% AB serum at 37°C + 5% CO_2_ in the presence and absence of NAC (10 mM) or L-GSH (10 and 20 *μ*M). Infected neutrophils were terminated at 1 hour and 24 hours after infection to determine the intracellular survival of H37Rv. Graph shows mean ± standard error.(a): Represents foldincrease in intracellular growth of H37Rv in neutrophils treated with NAC and L-GSH. (b): Determination of neutrophil viability following infection with H37Rv and treatment with NAC and L-GSH by quantifying DAPI stained cells (*n* = 5).

We tested the effects of GSH-enhancing agents in improving the functions of neutrophils to inhibit the growth of *M. tb. *This was achieved by determining the intracellular survival of H37Rv, a virulent laboratory strain of *M. tb* inside NAC/L-GSH-treated human neutrophils. We observed a 4-fold increase in the intracellular growth of H37Rv inside unstimulated neutrophils (Figure 1(a)). Treatment of neutrophils with L-GSH at 10 and 20 *μ*M concentrations resulted in a decrease in the foldgrowth of *M. tb* (Figure 1(a)). In fact, we observed maximum inhibition in the growth of H37Rv in neutrophils that were treated with L-GSH, and this is in contrast to untreated neutrophils in which there was a 4-fold increase in the growth of intracellular *M. tb* (Figure 1(a)). Interestingly, treatment of neutrophils with the GSH-precursor, NAC, at 50x higher concentration compared to L-GSH did not reduce the growth of *M. tb,* but instead we observed a 6-fold increase in the intracellular growth of H37Rv (Figure 1(a)). These results indicate that an optimal increase in the intracellular levels of GSH in neutrophils will lead to inhibition in the growth of *M. tb*. Treatment of H37Rv-infected neutrophils with GSH-enhancing agents did not result in any loss in the viability of neutrophils as evident from the quantification of DAPI stained cells (Figure 1(b)).

### 3.2. Assay of GSH Levels in Neutrophils

**Figure 2:**
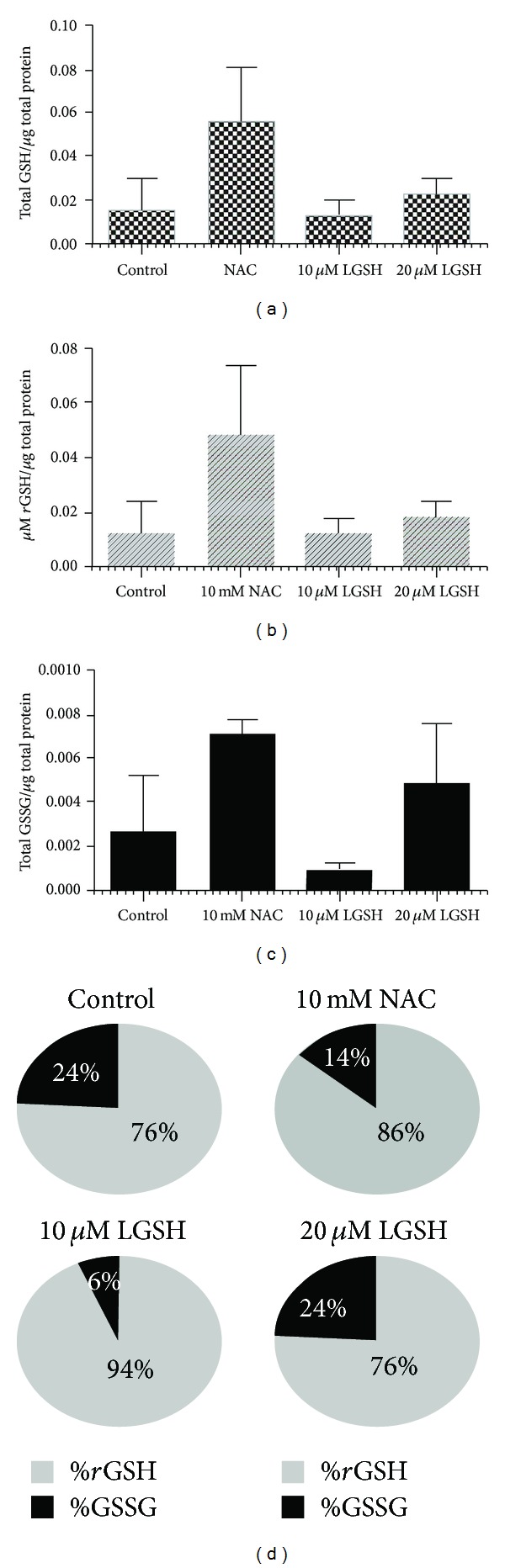
*GSH measurements in uninfected neutrophils cultured in the presence and absence of NAC or L-GSH (n* = 5*).* GSH levels were measured in isolated neutrophils from healthy subjects that were treated with GSH-enhancing agents such as NAC and L-GSH, by spectrophotometry using an assay kit. Briefly, neutrophils (3 × 10^5^) were lysed at 24 hours after treatment with ice cold 5% 5-sulfosalicylic acid dehydrate solution. Supernatants collected after centrifugation were analyzed for total GSH (a) and GSSG (c) as per manufacturer's instructions. *r*GSH was calculated by subtracting measured GSSG concentrations from the measured total GSH concentrations (b). All GSH measurements were normalized with total protein levels. Proteins in the cell lysates of neutrophils were measured by Bradford's method using a Coomassie protein assay reagent. (d) illustrates ratio of *r*GSH to GSSG expressed as percentage of total GSH in neutrophil lysates. Graph shows mean ± standard error.

**Figure 3:**
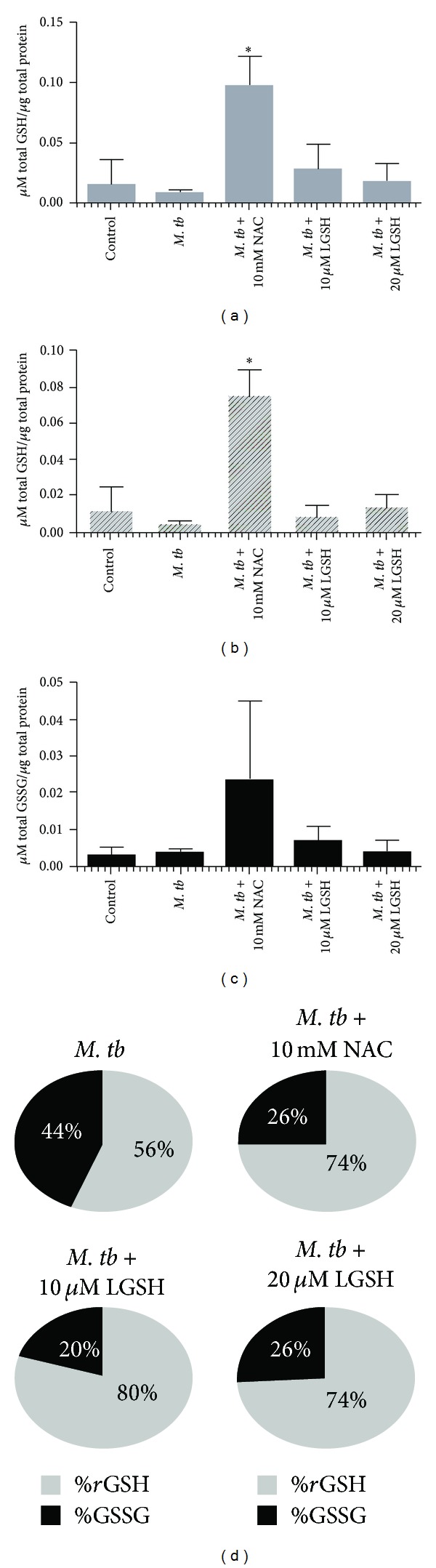
*GSH measurements in neutrophils infected with H37Rv and treated with NAC and L-GSH (n* = 5*).* GSH levels were measured in isolated neutrophils from healthy subjects that were infected *in vitro *with H37Rv and treated with GSH-enhancing agents such as NAC and L-GSH, by spectrophotometry using an assay kit. Briefly, H37Rv-infected neutrophils (3 × 10^5^) were lysed at 24 hours after infection with ice cold 5% 5-sulfosalicylic acid dehydrate solution. Supernatants collected after centrifugation were analyzed for total GSH ((a)*P ≤ 0.05) and GSSG (c) as per manufacturer's instructions. *r*GSH ((b)*P ≤ 0.05) was calculated by subtracting measured GSSG concentrations from the measured total GSH concentrations. All GSH measurements were normalized with total protein levels. Proteins in the cell lysates of neutrophils were measured by Bradford's method using a Coomassie protein assay reagent. (d) illustrates ratio of *r*GSH to GSSG expressed as percentage of total GSH in neutrophil lysates. Graph shows mean ± standard error.

We determined the ability of NAC and L-GSH to enhance the intracellular levels of GSH in uninfected neutrophils and restore the levels of GSH in H37Rv-infected neutrophils by spectrophotometry. We used an assay kit from Arbor Assays. We observed that treatment of uninfected neutrophils with NAC resulted in 3-fold increase in the levels of both total GSH and *r*GSH (Figure 2). L-GSH treatment did not result in any increase in the levels of both total GSH and *r*GSH in uninfected neutrophils (Figure 2). Importantly, H37Rv infection resulted in a 20% decrease in the levels of total GSH in neutrophils (Figure 3(a)). The percentages of *r*GSH versus GSSG in H37Rv-infected neutrophils was 56% and 44%, respectively (Figure 3(d)). Whereas, the percentage of *r*GSH and GSSG in uninfected neutrophils were 76% and 24%, respectively (Figure 2(d)). Our results signify that *M. tb* infection leads to decreased levels of *r*GSH, the functional form of GSH. Treatment of H37Rv-infected neutrophils with 10 mM NAC resulted in a 10-fold increase in the intracellular levels of total GSH (Figure 3(a)). Treatment of H37Rv-infected neutrophils with L-GSH (10 and 20 *μ*M concentration) resulted in restoration in the intracellular levels of both total GSH and *r*GSH to similar levels as uninfected neutrophils (Figures 3(a) and 3(b)). Treatment of H37Rv-infected neutrophils with GSH-enhancing agents (NAC and L-GSH) also resulted in reversal of GSSG and *r*GSH ratio similar to the percentage in uninfected neutrophils (Figure 3(d)). Our results indicate that *M. tb* infection decreases the levels of GSH in neutrophils possibly by inducing oxidative stress (as evident from the increased percentage of GSSG) and treatment of *M. tb*-infected neutrophils with GSH-enhancing agents restores the intracellular levels of both total GSH and *r*GSH.

### 3.3. Determination of Free Radicals Levels in Neutrophils

**Figure 4:**
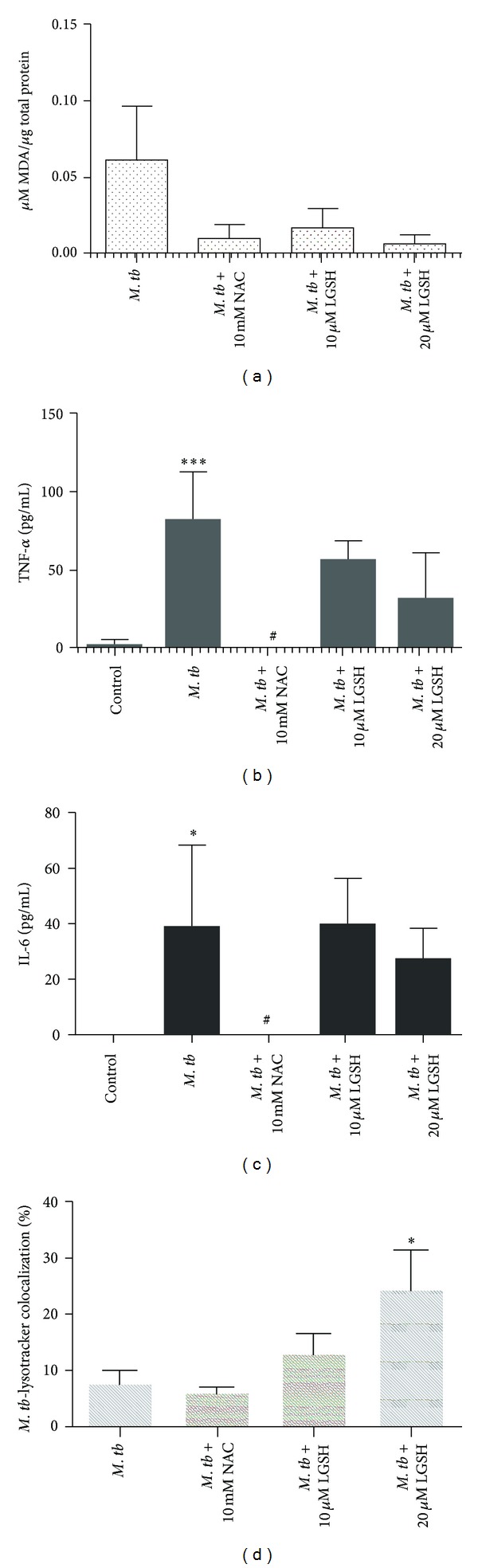
*Assay of MDA in neutrophil lysates at 24 hours after infection with H37Rv (n* = 5*).* Free radical levels in neutrophil lysates were determined by measuring the levels of MDA using a colorimetric assay kit from Cayman. These elevated MDA concentrations are indicative of elevated free radical concentrations. Treatment with either NAC or L*-*GSH reduced the amount of MDA. (b) and (c): Cytokine measurements performed in supernatants derived from H37Rv-infected neutrophils. Levels of TNF-*α* (b) and IL-6 4(c) in neutrophil supernatants were measured by ELISA. (d): Determination of phagosome acidification by quantifying the colocalization of FITC-labeled H37Rv with lysotracker: neutrophils were stained with lysotracker and infected with FITC labeled H37Rv. Infected neutrophils were incubated for 24 hours on sterile glass cover slips in the presence and absence of GSH-enhancing agents. Neutrophils were fixed with 3.8% PFA in PBS for 30 minutes, and the coverslips were mounted on a clean glass slide using DAPI. Slides were then viewed using an inverted fluorescent microscope for the colocalization of FITC-labeled H37Rv with lysotracker which is indicative of phagosome acidification. Images were obtained using an integrated digital camera and analyzed using ImageJ. ***P ≤ 0.001; ^#^ *P* ≤ 0.05. Graph shows mean ± standard error.

We determined the inverse correlation of GSH levels and ROI by quantifying the levels of free radicals in H37Rv-infected neutrophils. We observed that infection of neutrophils with H37Rv resulted in a 3-fold increase in the production of free radicals compared to the uninfected neutrophils (Figure 4(a)). NAC and L-GSH treatments led to a noticeable decrease in the levels of free radicals. The decreased levels were similar to the uninfected neutrophils (Figure 4(a)). Maximum decrease in the levels of free radicals was observed when H37Rv-infected neutrophils were treated with 20 *μ*M L-GSH (Figure 4(a)). Our results highlight the ability of* M. tb* infection to induce neutrophils to produce free radicals leading to a decrease in the levels of *r*GSH.

### 3.4. TNF-*α* Assay in Neutrophil Supernatants

 H37Rv infection of neutrophils resulted in more than 28-fold increase in the production of TNF-*α* compared to uninfected neutrophils (Figure 4(b)). Treatment of H37Rv-infected neutrophils with NAC (10 mM) caused a dramatic decrease in the production of TNF-*α*. In fact, the TNF-*α* levels were undetectable in supernatants collected from H37Rv-infected + NAC-treated neutrophils (Figure 4(b)). These results indicate that NAC is capable of completely diminishing the production of TNF-*α* by H37Rv-infected neutrophils. Treatment of H37Rv-infected neutrophils with 10 and 20 *μ*M L-GSH did not result in any decrease in the production of TNF-*α* compared to untreated H37Rv-infected neutrophils (Figure 4(b)). 

### 3.5. Assay of IL-6 in Neutrophil Supernatants

H37Rv infection of neutrophils resulted in more than 38-fold increase in the production of IL-6 compared to uninfected neutrophils (Figure 4(c)). Treatment of H37Rv-infected neutrophils with NAC (10 mM) caused a striking decrease in the production of IL-6 (Figure 4(c)). IL-6 levels were undetectable in supernatants collected from H37Rv-infected + NAC-treated neutrophils. Treatment of H37Rv-infected neutrophils with L-GSH resulted did not result in any significant decrease in the production of IL-6 compared to untreated H37Rv-infected neutrophils (Figure 4(c)). These results indicate that NAC treatment, at millimolar concentrations, is capable of completely diminishing the production of both IL-6 and TNF-*α* by H37Rv-infected neutrophils (Figures 4(b) and 4(c)).

### 3.6. Examining the Acidification of *M. tb* Containing Phagosomes by Quantifying the Colocalization of FITC-Labeled H37Rv with Lysotracker

**Figure 5:**
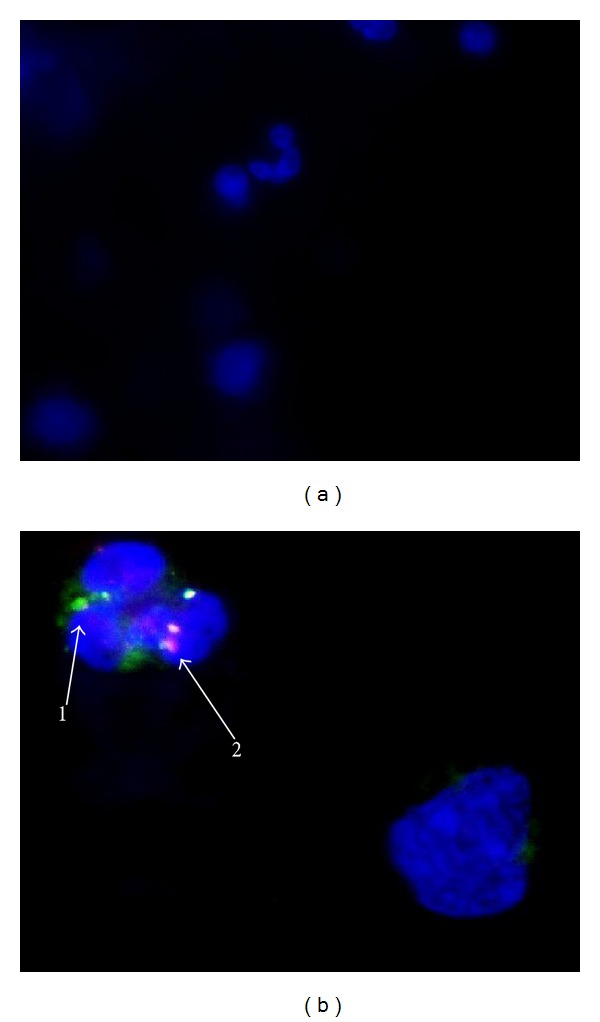
Fluorescent microscopic images of neutrophils obtained using an integrated digital camera. (a): DAPI staining of neutrophils (40x oil immersion). (b): DAPI staining of neutrophils, FITC stained H37Rv colocalizing with the lysotracker-labeled acidified compartment (100x oil immersion). Label 1 represents green fluorescent-labeled H37Rv in nonacidified phagosomal compartment. Label 2 represents colocalization of green fluorescent-labeled H37Rv with lysotracker, indicating acidification of H37Rv-containing phagosome.

We examined the effects of GSH on increasing the acidification of phagosomes containing *M. tb* by quantifying the colocalization of FITC-labeled H37Rv with lysotracker trapped in the acidified compartments in the neutrophils (Figure 5). We observed that treatment of H37Rv-infected neutrophils with 10 *μ*M L-GSH resulted in 40% increase in the colocalization of FITC-labeled H37Rv with lysotracker (Figure 4(d)). Interestingly, treatment of H37Rv-infected neutrophils with 20 *μ*M L-GSH resulted in a significant and 2-fold increase in the colocalization of FITC-labeled H37Rv with lysotracker in comparison to infected untreated category (Figure 4(d)). Treatment of H37Rv-infected neutrophils with NAC (10 mM) did not result in any increase in the acidification of *M. tb *containing phagosomes (Figure 4(d)). These results indicate that optimal levels of GSH promote fusion between phagosomes containing *M. tb *and lysosomes in neutrophils.

## 4. Discussion

Mycobacteria do not synthesize GSH. Rather, they produce mycothiol in millimolar amounts [15]. We have observed that the virulent laboratory strain of *M. tb*, H37Rv is sensitive to GSH at physiological concentrations (5 mM) when grown *in vitro* [15]. The mechanism of antimycobacterial activity of GSH could very well be due to a redox imbalance in a bacterium containing an alternative thiol, such as mycothiol, that regulates reduction/oxidation activity [15]. We also found that enhancing the levels of GSH in human macrophages by treatment with NAC (10 mM) and L-GSH (10 *μ*M) resulted in inhibition in the growth of intracellular H37Rv [16]. Thus, GSH has direct antimycobacterial activity, functioning as an effector molecule in innate defense against *M. tb *infection [15, 17–19]. These results unfold a novel and potentially important innate defense mechanism adopted by human macrophages to control *M. tb *infection [15, 17–19]. Notably, we observed that L-GSH is able to inhibit the growth of *M. tb *inside human macrophages at a log lower concentration in comparison to NAC [16]. Our recent studies indicate that GSH in combination with cytokines such as IL-2 and IL-12 enhances the functional activity of NK cells to inhibit the growth of *M. tb *inside human monocytes [14]. Importantly, data from our latest studies indicate that GSH activates the functions of T lymphocytes to control *M. tb *infection inside human monocytes [13]. Finally, we demonstrated that GSH levels are significantly compromised in peripheral blood mononuclear cells and red blood cells isolated from individuals with active pulmonary TB [20]. This decrease correlated with increased production of pro-inflammatory cytokines and enhanced growth of *M. tb*. All these observations support the fact that GSH controls *M. tb *infection by functioning as an antimycobacterial agent as well as by enhancing the functions of immune cells [20].

Neutrophils are less well studied than other immune cells that are involved in defense against *M. tb* infection, and much of the available data is controversial. This is probably in part because of inherent difficulties in working with these cells. A key role for neutrophils is demonstrated in host defense against *M. tb* infection. In particular, circulating neutrophils become activated and are recruited to lungs early during infection. Their role in host defense against *M. tb *is supported by studies showing that depletion of neutrophils before i.v. challenge with *M. tb *compromises the immune responses against mycobacterial infection [2, 4–6, 21].

We explored the effects of GSH-enhancing agents in improving the functions of isolated neutrophils from healthy subjects to inhibit the growth of *M. tb*. We observed that treatment of H37Rv-infected neutrophils with L-GSH resulted in stasis in the growth of H37Rv. The inhibition in the growth of H37Rv inside L-GSH-treated neutrophils was accompanied by restoration in the levels of both free GSH and *r*GSH and notable decrease in the levels of free radicals. Interestingly, L-GSH treatment of H37Rv-infected neutrophils did not alter the synthesis of IL-6 and TNF-*α*. Importantly, we observed an increased acidification of H37Rv containing phagosomes in neutrophils that were treated with L-GSH, highlighting a potential underlying mechanism that could contribute to the inhibition of *M. tb* growth in L-GSH-treated neutrophils. TNF-**α** has been shown to play a major role in activating various effector mechanisms inside neutrophils and macrophages leading to inhibition in the growth of *M. tb* [22–26]. Furthermore, TNF-**α** is essential for the formation and maintenance of granuloma which is critical for restricting the spread of *M. tb* infection [22–27]. Human studies demonstrate that patients receiving TNF-*α* inhibitors for chronic inflammatory diseases such as rheumatoid arthritis and Crohnrs disease can develop active TB due to reactivation of latent *M. tb* infection [12, 22, 28–30], further highlighting the significance of TNF-*α* in protection against *M. tb* infection. IL-6 is also considered important for mediating control of *M. tb* infection [31]. In fact, it has been reported that IL-6 blockade resulted in increased susceptibility to *M. tb* infection in IL-6-deficient mice [32] and anti-IL-6 antibody-treated mice [33].

Recently, Okada et al have examined whether IL-6R blockade influences the course of *M. tb* infection in BALB/c mice and compared the effects of IL-6 blockade with those of TNF-**α** blockade in the same experiments performed at the same time [34]. Their findings demonstrate that blocking IL-6 receptor by treatment with anti-IL-6R antibodies increases the susceptibility of these mice to *M. tb* infection but that this increase is far less marked than that induced by anti-TNF-**α** antibody treatment indicating that the involvement of IL-6 in protection against *M. tb* infection is much less important than that of TNF-**α**[34]. Our results signify that increased acidification of *M. tb* containing phagosomes leading to the inhibition in the growth of *M. tb* in neutrophils is dependent on the presence of TNF-*α* and IL-6. However, GSH enhancement in neutrophils following NAC treatment was not accompanied by inhibition in the growth of *M. tb*. Surprisingly, we observed a several-fold increase in the survival of *M. tb*. NAC treatment resulted in significant downregulation in the levels of TNF-*α* and IL-6. In fact, the levels of TNF-*α* and IL-6 were undetectable in NAC-treated neutrophils, and this may explain the reason for poor localization of *M. tb* in acidified lysosomes (Figures 4(b), 4(c) and 4(d)). Our studies also reveal that free radicals may not directly contribute to the control of *M. tb* infection inside the neutrophils (Figure 4(a)).

Increased production of TNF-*α* and IL-6 accompanied by insufficient levels of GSH can generate excess free radicals that may be disadvantageous to the host because they not only cause acute-phase events, such as fever, but also mediate cachexia, hemorrhagic necrosis, and lethal shock [35–38]. Necrosis and hypoxia are key pathological features of human TB lesion and are thought to favor survival of persistent bacteria [39]. Increased production of free radicals can induce both structural and functional changes in the internal membranes of the organelles in the cells leading to defects in the fusion and trafficking of intracellular vesicles.

Therefore, the presence of optimal levels of TNF-*α* in conjunction with GSH will not only preserve the integrity of intracellular vesicular membranes but will also enhance the fusion between phagosomes and lysosomes, thereby, inhibiting the multiplication of *M. tb*.

**Figure 6:**
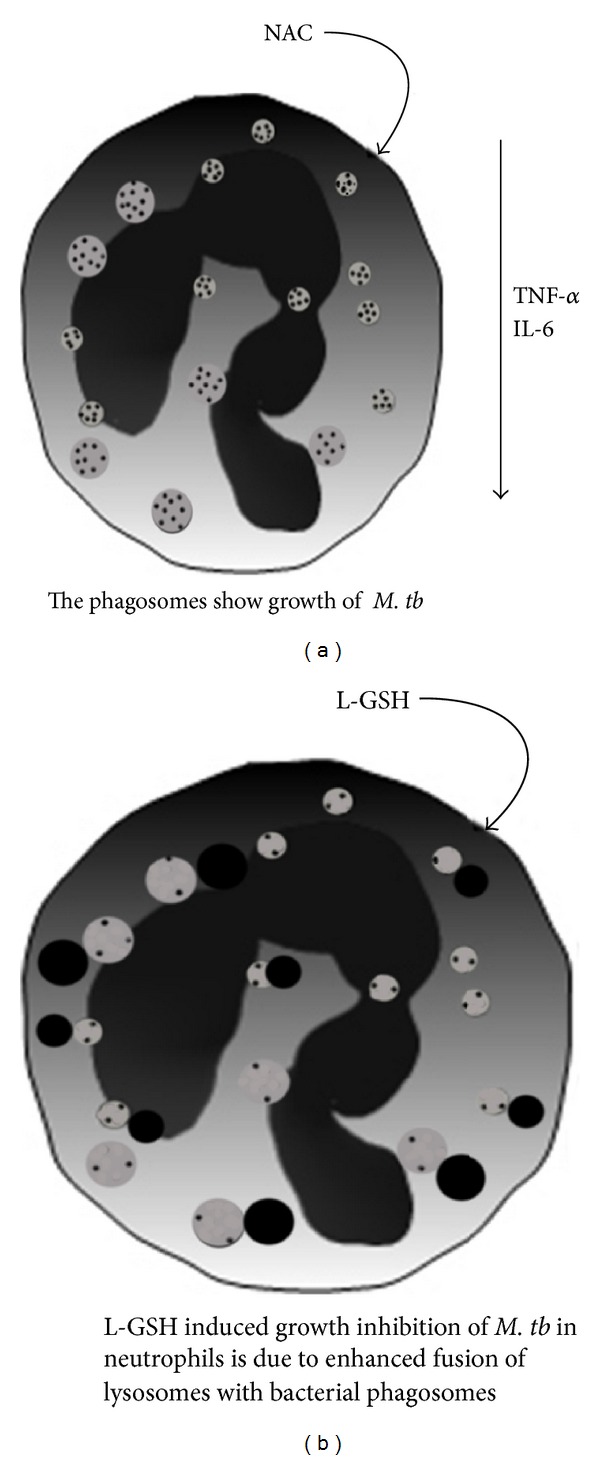
Model illustrating the underlying mechanisms that are responsible for growth of *M. tb *in NAC-treated neutrophils (a) and inhibition in the growth of *M. tb *in L-GSH-treated neutrophils (b).

Our studies emphasize the need for a fine balance in the levels of GSH and proinflammatory cytokines to effectively inhibit the intracellular growth of *M. tb* inside the neutrophils. Conversely, if this delicate balance is disturbed, then it will lead to enhanced growth of *M. tb*. This is evident from the results of our studies using NAC (10 mM) treatment to enhance the levels of GSH in infected neutrophils. This phenomenon may explain why L*-*GSH at lower concentrations than NAC is more effective in inhibiting the growth of *M. tb* in neutrophils. Supplementing with an L*-*GSH formulation provides complete *r*GSH molecules to cells, circumventing the enzymatic pathway responsible for *r*GSH production, without the requirement for the cell to construct the tripeptide [40]. This may also explain why treatment with L*-*GSH seems to raise the ratio of *r*GSH to GSSG at much lower concentrations than NAC, as cells treated with NAC will have to produce new molecules of *r*GSH utilizing their own enzymatic machinery. To summarize, our results indicate that GSH mediates control of *M. tb* infection inside human neutrophils. One mechanism by which GSH inhibits the growth of *M. tb* inside neutrophils is by decreasing the levels of free radicals, thereby promoting fusion between *M. tb*-containing phagosomes with lysosomes, an important effector mechanism that is important for inhibiting the growth of *M. tb*. Our results demonstrate a pattern of oxidative stress brought on by *M. tb* infection, which decreases *r*GSH and impairs the intracellular killing of *M. tb *in neutrophils (Figure 6(a)). By supplementing *r*GSH, we were able to mitigate the production of ROI and enhance the acidification of phagosomes, thereby improving the ability of neutrophils to kill *M. tb *intracellularly (Figure 6(b)). The results from our 10 *μ*M LGSH treatment demonstrate the maximum inhibition of *M. tb *growth (Figure 1(b)); however, results from 20 *μ*M LGSH treatment show the maximum reduction in free radicals and maximum phagosome-lysosome fusion (Figures 4(a) and 4(d)). These results imply that there is an additional mechanism for the control of *M. tb* growth within neutrophils that can be modulated by *r*GSH. Future experiments should attempt to identify these mechanisms.
